# A dominant clonal lineage of *Streptococcus uberis* in cattle in Germany

**DOI:** 10.1007/s10482-022-01740-w

**Published:** 2022-04-30

**Authors:** Linda Fenske, Irene Noll, Jochen Blom, Christa Ewers, Torsten Semmler, Ahmad Fawzy, Tobias Eisenberg

**Affiliations:** 1 Hessian State Laboratory, Department of Veterinary Medicine, Giessen, Germany; 2 Bioinformatics and Systems Biology, Justus Liebig University, Giessen, Germany; 3 Regional Council of Gießen, Wetzlar, Germany; 4 Institute of Hygiene and Infectious Diseases of Animals, Justus Liebig University, Giessen, Germany; 5 NG1 Microbial Genomics, Robert Koch Institute (RKI), Berlin, Germany; 6 Department of Medicine and Infectious Diseases, Faculty of Veterinary Medicine, Cairo University, Giza, Egypt

**Keywords:** Bovine mastitis, Comparative genomics, Multilocus sequence typing, Resistance, *Streptococcus uberis*, Prophage regions, Virulence

## Abstract

Bovine mastitis causes enormous economic losses in the dairy industry with *Streptococcus uberis* as one of the most common bacterial pathogens causing clinical and subclinical variations. In most cases mastitis can be cured by intramammary administration of antimicrobial agents. However, the severity of the clinical manifestations can vary greatly from mild to severe symtoms. In this study, a comparative genomic analysis of 24 *S. uberis* isolates from three dairy farms in Germany, affected by different courses of infection was conducted. While there were sporadic mild infections in farm A and B, a large number of infections were observed within a very short period of time in farm C. The comparison of virulence genes, antimicrobial resistance genes and prophage regions revealed no features that might be responsible for this severe course. However, almost all isolates from farm C showed the same, novel MLST profile (ST1373), thus a clonal outbreak cannot be excluded, whereby the actual reason for the particular virulence remains unknown. This study demonstrates the importance of extensive metagenomic studies, including the host genomes and the environment, to gain further evidence on the pathogenicity of *S. uberis*.

**Supplementary Information:**

The online version contains supplementary material available at 10.1007/s10482-022-01740-w.

## Introduction

Bovine mastitis causes enormous economic losses in the dairy industry (Hogeveen et al. 2011). It is estimated that the loss amounts to approximately 124 Euros per cow annually, resulting in annual losses of 500 million Euros in Germany and up to 125 billion Euros worldwide (Kabelitz et al. 2021). Bovine mastitis is defined as an inflammation of the udder, which is characterised by an increase in somatic cell count (SCC) and typically by the presence of udder pathogens. In case of subclinical mastitis, there are no clinical signs beside SCC elevations, whereby clinical mastitis is characterised by a visibly altered milk or udder, which can reach various degrees of severity (GVA 2012). If no udder pathogens are detected and there are subclinical or clinical findings, it is a matter of non-specific mastitis. Mastitis can be caused by a wide variety of pathogens, e.g. bacteria, yeasts or algae (Bradley 2002) and some viral pathogens aswell (Wellenberg et al. 2002). *Streptococcus uberis*, a Gram-positive, catalase-negative member of the family *Streptococcaceae* with a genome size of about 1.8–2.3 Mbp is one of the most important environmentally associated pathogens responsible for bovine udder infections (Whiley and Hardie 2015). Mastitis caused by *S. uberis* in particular can be both subclinical and clinical, the latter often causing mild to severe visible signs of inflammation (Zadoks et al. 2003). It is not yet certain whether these different courses of infection are solely based on the genetic diversity of involved bacterial strains or if other (e.g. environmental, immune) factors also play a role (Günther et al. 2016). In general, *S. uberis*, which is also known to be shed with bovine faeces, normally does not spread from udder to udder (non-contagious). Furthermore, severe infections with this pathogen by a lymphogenic or haematogenic route to extra-mammary tissues are rarely observed (Thomas et al. 1994). Occasionally, *S. uberis* seems to induce a contagious type of mastitis as suggested by recent clinical case series (Davies et al. 2016; Wente et al. 2019). In this regard, this bacterium is able to adapt to different environmental conditions, body sites and also stress situations with different gene expressions (Ward et al. 2009). Likewise, antimicrobial and in particular *β*-lactam resistant strains of *S. uberis* have been observed more frequently in the recent past, which might have been induced by certain substitutions in the sequences of the penicillin binding proteins (pbp) leading to increased resistance to oxacillin (McDougall et al. 2020). Although several strains have been typed using multilocus sequence typing (MLST), only a few fully sequenced genomes exists to this date. Nevertheless, the number of sequenced draft genomes continues to increase and studies in this field are becoming more and more relevant (Hossain et al. 2015; Vezina et al. 2021). Currently, 69 genomes can be found in the NCBI database. But, despite extensive research, prophylactic vaccination against *S. uberis* is still under debate, because little is known about the interaction between *S. uberis* and its host and generally knowledge about the pathogen’s genomic traits is still scarce (Hossain et al. 2015).

In this study, we conducted a comparative genomic analysis using 24 *S. uberis* isolates, obtained from 3 dairy farms in Germany, that were experiencing different courses of infection. The results could help to extend the insight into the molecular epidemiology of this important pathogen.

## Material and methods

### Cattle herds under study

For this study aseptically drawn milk samples were sent to the laboratory for cyto-bacteriological investigation (CBI). A total of 24 *S. uberis* strains collected from 3 different farms in Germany between 2016 and 2019 were included in the study. All three herds on the farms are located in the same surrounding and were characterised by the same breed, age ranges of sick animals, usage of cows, hygienic conditions, used sanitisers and milking practice (Suppl. Tab 1). *S. uberis* infections represented the predominant mastitis pathogen, however, some mixed infections with coagulase-negative staphylococci were detected, but not considered for sampling. No *Mycoplasmopsis bovis* infections were detected by PCR in pooled samples (data not shown). The spread of *S. uberis* infections differed between the farms. While there were sporadic mild infections in farm A and B (group A and B), a large number of infections were observed within a period of eight weeks in farm C (group C). The severity of the infection in farm C was also much more pronounced compared to farm A and farm B and included mostly severe inflammations of the udder and fever. Various antimicrobial treatment schemes (data not shown) ended up in no or only short-term clinical improvement, although the streptococci detected in the antimicrobial susceptibility test were reported to be sensitive to all the substances used (s. below). Although *S. uberis* is usually an environmental pathogen, in the present case it resembled a contagious course of infection that is spreading from cow to cow. About 30 lactating cows had to be slaughtered, because of the severity of mastitis despite antibiotic treatment. At the time of investigation on farm C, 106 animals were lactating. Milk samples from 69 cows hav been taken and were submitted to the laboratory for CBI. In 32 of the animals examined, *S. uberis* in connection with an increased somatic cell count could be detected in at least one quarter of the udder. Fourteen of these strains were selected for further investigations in this study.

### Microbiological culture and identification of mastitis pathogens including *S. uberis*

Milk samples were directly streaked on inhouse prepared Columbia agar with 5% cattle blood and esculin (ingredients provided by Oxoid, Wesel, Germany) and cultivated using aerobic atmosphere conditions for 48 h at 37 °C. Yeast growth was investigated using a Sabouraud glucose agar with gentamicin and chloramphenicol (Oxoid) at 30 °C in samples containing an SCC of more than 3 × 10^6^ somatic cells/mL. Isolates were further evaluated using Gram’s staining and matrix assisted laser desorption/ionisation—time of flight mass spectrometry (MALDI-TOF MS; microflex LT Mass Spectrometer, MALDI Biotyper™; Bruker Daltonik, Bremen, Germany) using the direct smear method in sample preparation and the commercial MALDI-Biotyper database (MBT 8468; Bruker Daltonik).

### Antimicrobial susceptibility testing

Antimicrobial susceptibility testing (AST) was carried out using broth microdilution testing. Briefly, a commercially available panel layout for mastitis treatment (Micronaut/Bruker according to guidelines of the research group antimicrobial resistance of the German Veterinary Society DVG) was used. In this layout, 11 different antimicrobials were employed [(ranges given in µg mL^−1^); penicillin (0.063–4), oxacillin (0.063–2), amoxicillin/clavulanic acid (0.031/0.063–8/16), ampicillin (0.125–8), cefazolin (0.5–16), cefoperazon (0.25–4), cefquinome (1–16), kanamycin/cephalexin (0.031–2), marbofloxacin (0.016–2), erythromycin (0.125–4) and pirlimycine (0.25/4.75–2/38)]. Resulting MIC values were interpreted as sensitive, resistant and intermediate resistant based on clinical breakpoints according to CLSI VET01/VET01S (5th ed.) for broth microdilution testing. *Escherichia coli* ATCC 25,922, *Pseudomonas aeruginosa* ATCC 27,853, *Enterococcus faecalis* ATCC 29,212 and *Staphylococcus aureus* ATCC 29,213 served as quality control strains.

### Sequencing and bioinformatic processing

Next-generation-sequencing (NGS) was carried out with 24 representative *S. uberis* isolates from dairy farms in Germany. From randomly chosen control farms, A and B, 10 isolates (each n = 5) were selected, whereas the remaining *S. uberis* isolates were included from farm C (group C, n = 14). Genomic DNA was extracted from a 72 h bacterial culture and sequencing was employed using Illumina MiSeq and Illumina NextSeq 150 bp paired-end sequencing with an obtained coverage of > 70x (Table 1). Raw reads were used for de novo assembly into contiguous sequences and subsequently into scaffolds using SPAdes (Bankevich et al. 2012). Assemblies were annotated using Bakta (Schwengers et al. 2021). MLST profiles were checked with PubMLST (pubmed.org/suberis). For clonal complex assignment of isolates and classification of sequence types in the overall population, goeBURST was used (Feil et al. 2004). For determination of the pan/core genome, as well as for orthologous genes, EDGAR 3.0 was used (Dieckmann et al. 2021). The PHASTER webtool was used to identify prophage sequences within the draft genomes (Arndt et al. 2016). ABRicate, with an individual gene set of *S. uberis* putative pathogenicity factors described in the literature, was used for virulence and resistance gene analysis (https://github.com/tseemann/abricate). As reference for all analyses the genome sequences of the strains 0140J (accession number AM946015)—which has been isolated from milk obtained from clinical case of bovine mastitis in 1972 and sequenced in 2009 as a typical virulent UK isolate pathogenic to lactating as well as non-lactating bovine mammary glands (Ward et al. 2009)—and EF20 (accession number JANW01) as an nonvirulent strain according to Hossain et al. 2015, were used. To gain insight into possible *β*-lactam antibiotic resistance, the genome of strain 0140J was first searched for penicillin-binding proteins. EDGAR was then used to search for orthologues of these genes in all the draft genomes, and a subsequent BLAST search was done to locate substitutions within the amino acid sequences of these genes (Altschul et al. 1990).

**Table 1:** Assembly statistics of 24 *Streptococcus uberis* isolates

Isolate	Sequencing platform	No. of contigs	N50	Average coverage range
Su-1	Illumina NextSeq 500	28	159,211	275–2463
Su-2	Illumina MiSeq	16	175,018	184–1665
Su-3	Illumina MiSeq	15	457,442	134–1210
Su-4	Illumina NextSeq 500	31	197,492	83–3848
Su-5	Illumina MiSeq	14	206,517	130–1164
Su-6	Illumina MiSeq	24	229,596	100–932
Su-7	Illumina NextSeq 500	23	313,412	138–3145
Su-8	Illumina MiSeq	37	123,181	202–3331
Su-9	Illumina NextSeq 500	21	255,232	129–3911
Su-10	Illumina NextSeq 500	38	206,273	95–4030
Su-11	Illumina MiSeq	13	436,510	160–1445
Su-12	Illumina MiSeq	12	204,844	166–1565
Su-13	Illumina MiSeq	15	439,515	74–1330
Su-14	Illumina MiSeq	12	1,031,011	92–1363
Su-15	Illumina MiSeq	13	1,030,881	265–3465
Su-16	Illumina MiSeq	13	1,030,845	458–3633
Su-17	Illumina MiSeq	12	439,836	406–4468
Su-18	Illumina MiSeq	49	79,688	56–3424
Su-19	Illumina MiSeq	12	439,511	206–1692
Su-20	Illumina MiSeq	18	320,790	114–2080
Su-21	Illumina MiSeq	21	187,067	83–1150
Su-22	Illumina MiSeq	12	439,515	121–2124
Su-23	Illumina MiSeq	13	436,512	474–4427
Su-24	Illumina MiSeq	16	439,517	352–5571

## Results

### Microbiological culture results

Herds under study showed a similar distribution of mastitis pathogens with *S. uberis* found in several quarters. It was the only relevant microorganism that was mostly found. In farm A, B and C 6/48 (12.5 %), 17/140 (12.1 %) and 48/252 (19.0 %) quarters were positive for this microorganism, respectively. All *S. uberis* isolates were confirmed by MALDI-TOF MS and a subset of 24 isolates was chosen for WGS analysis based on monobacterial infections and a high semi-quantitative count (> 200 colonies per quarter) of *S. uberis* together with high SCC above 1 × 10^6^ cells/mL. Samples with mixed infections or possible contamination were not taken into account.

### Antimicrobial susceptibility testing

Based on AST as conducted by broth microdilution testing, all strains from this study were susceptible to all antimicrobials tested (Table 2).

**Table 2:** Representative *Streptococcus uberis* AST phenotype

Parameter	Interpretation result	MIC values
Amoxicillin/clavulanic acid	S	< = 4/2
Ampicillin	S	< = 4
Cefquinome	S	< = 1
Cefazolin	S	< = 4
Cefoperazon	S	< = 2
Erythromycin	S	< = 0.125
Kanamycin/cephalexin	S	< = 4/0.4
Marbofloxacin	S	= 0.5
Oxacillin	S	< = 1
Penicilling	S	< = 0.125
Pirlimycin	S	< = 1

### Sequencing

**Fig. 1:**
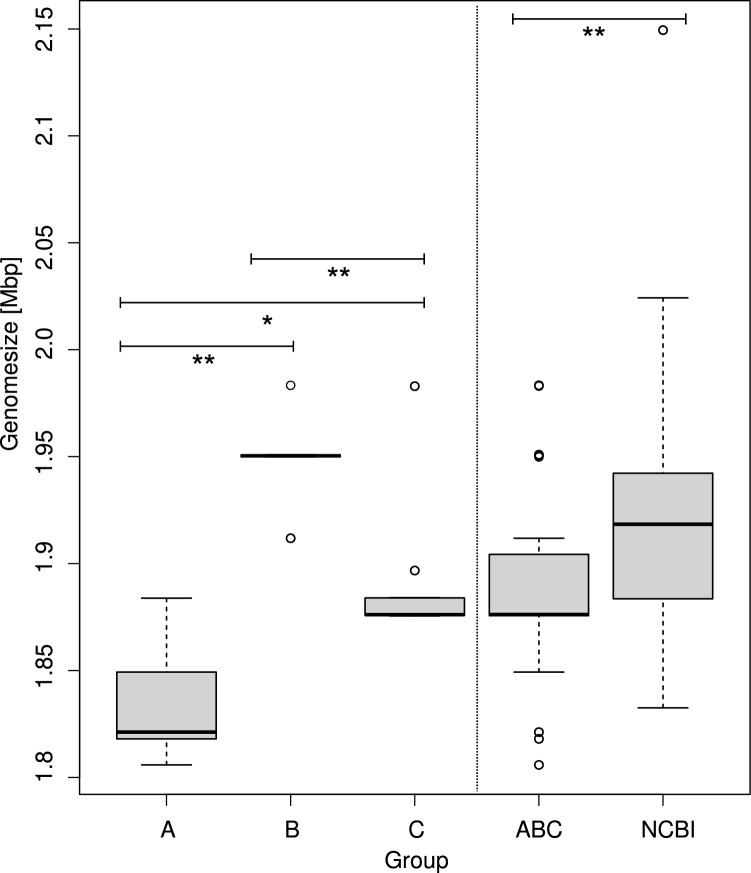
There is a high statistically significant difference (*p* < 0.01) between groups A and B and between groups B and C. Groups A and C differed significantly (*p* < 0.05). There is a high statistically signifant difference between all genomes currently found in the NBCI database and the total genome size of group A, B and C combined. Boxplot and calculation was created with R-Studio

The combined lengths of the assembled contigs ranging from 1,805,858 to 1,983,343 bp and the GC-content was in the range of 36.3–36.5 %, which is in line with the literature values (Table 3) (Whiley and Hardie 2015). In comparison, the GC content of all *S. uberis* genomes currently available in the NCBI database ranges between 36.1 and 36.8 % (data not shown). Isolates from farm A had a mean genome size of 1,835,641 bp (SB ± 31,282.29), whereas farm B isolates had a mean size of 1,949,274 bp (SD ± 25,325.31) and farm C isolates displayed a mean genome size of 1,886,202 bp (SD ± 28,483.27). With respect to genome size here was a high statistically significant difference (*p* < 0.01) between groups A and B and between groups B and C, whereas groups A and C differed significantly (*p* < 0.05). All genomes of *S. uberis* currently available in the NCBI database had a mean genome size of 1,917,489 bp (SD ± 53,593.74), which is high significant above the genomes examined in our study with a combined mean genome size of 1,888,809 (SD ± 46,408.21; *p* < 0.01; Fig. 1).

**Table 3:** Sequencing statistics of 24 *Streptococcus uberis* isolates plus the strains 0140 J and EF20

Isolate	sequence size (bp)	Number of contigs (> 500 bp)	Longest contig (bp)	Shortest contig (bp)	GC %
*Group A*					
Su-01	1,821,188	21	515,871	610	36.5
Su-02	1,818,057	12	1,002,414	800	36.5
Su-03	1,883,847	8	1,008,567	933	36.4
Su-04	1,849,257	21	318,687	933	36.5
Su-05	1,805,858	8	1,000,761	800	36.4
*Group B*					
Su-06	1,911,851	11	524,326	5 579	36.3
Su-07	1,950,395	14	659,959	891	36.4
Su-08	1,949,859	18	653,595	553	36.3
Su-09	1,950,920	9	1,070,520	933	36.4
Su-10	1,983,343	17	429,199	606	36.4
*Group C*					
Su-11	1,883,972	8	1,061,615	800	36.4
Su-12	1,875,458	8	1,032,662	548	36.3
Su-13	1,875,842	8	1,032,662	933	36.3
Su-14	1,875,689	9	1,031,954	933	36.3
Su-15	1,876,200	8	1,032,186	933	36.3
Su-16	1,876,102	11	1,031,729	508	36.3
Su-17	1,875,804	8	1,032,437	933	36.3
Su-18	1,982,968	50	922,189	508	36.4
Su-19	1,876,339	9	1,032,186	933	36.3
Su-20	1,896,764	15	1,035,846	567	36.4
Su-21	1,875,591	9	690,884	933	36.3
Su-22	1,875,809	8	1,032,566	933	36.3
Su-23	1,884,146	9	1,061,387	535	36.4
Su-24	1,876,146	8	1,032,828	933	36.3
*Reference strains*					
0140J	1,852,352	1	–	–	36.6
EF20	1,932,039	17	1,013,731	366	36.3

### Pan and core genome analysis

**Fig. 2:**
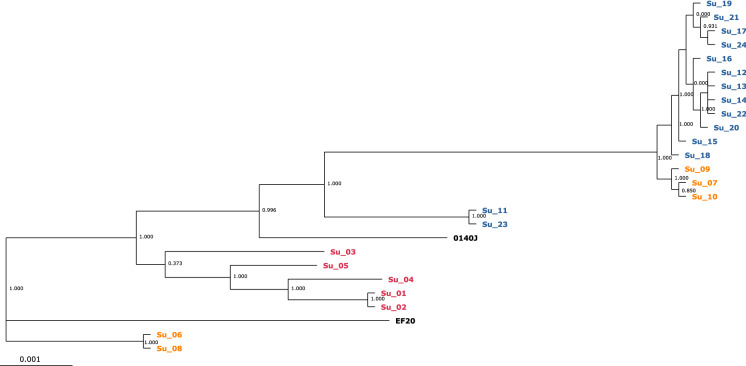
Core genome phylogenetic tree. Core genes of these genomes were computed in EDGAR 3.0 based on muscle alignment. An approximately-maximum-likelihood phylogenetic tree was calculated using the FastTree software. The core genome analysis was based on 1567 genes per genome in 24 strains plus the reference strains 0140J and EF20. The core had 489,494 amino acid residues/bp per genome, 12,726,844 in total. Bar, 0.001 nucleotide substitutions per site. The values at the branches are Shimodaira-Hasegawa support values. Isolates are highlighted according to group affiliation (Group A: red; Group B: orange; Group C: blue)

**Fig. 3:**
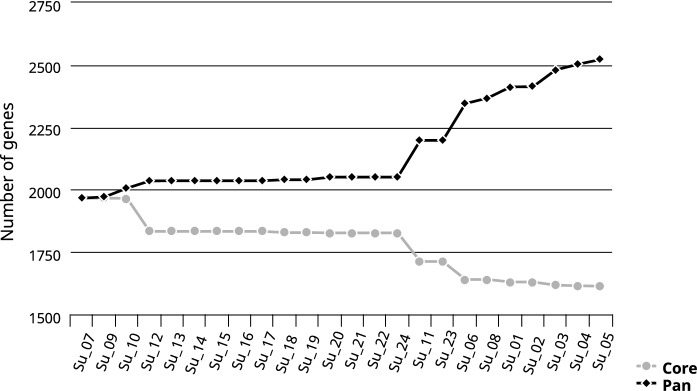
Pan versus core development plot to gain insight into the stability of the pan- and core-genome of the isolates. Results were obtained using EDGAR 3.0. Starting with the first contig, consecutive numbers for the core and pan genome size were calculated and plotted. The plot shows an increase of the pan-genome (black line) and a decrease of the core-genome (grey line) as more genomes are added

Examination of the gene content of all 24 isolates (referred to as Su-01—Su-24) resulted in a pan genome of 2508 genes, with a core genome of 1611 genes. With the addition of strain 0140J, the pan genome increased to 2551 genes, and with strain EF20, the pan genome increased to 2642 genes. To address the presumption of a clonal outbreak and to determine the exact relationships between the isolates, a phylogenetic tree based on the core genome was constructed using EDGAR 3.0 (Fig. 2). The topology of this tree was initially divided into three monophyletic groups. One group contained the isolates Su-06 and Su-08 from group B, the second clade only included the strain EF20, and the last branch contained all remaining isolates and the strain 0140J. Group A also formed a separate monophyletic group within the latter group. The isolates Su-11 and Su-23 from farm C form a group together with strain 0140J. All other isolates from group C clustered in the same clade. However, Su-07, Su-09 and Su-10 from group B were intermixed in this group. When looking at the development of the core and pan genome over the number of genomes, within group C, there is neither a significant increase in the pan genome nor a decrease in the core genome (Fig. 3). The increasing of the pan genome as well as the decreasing of the core genome showed when isolates from groups A and B are included.

**Fig. 4:**
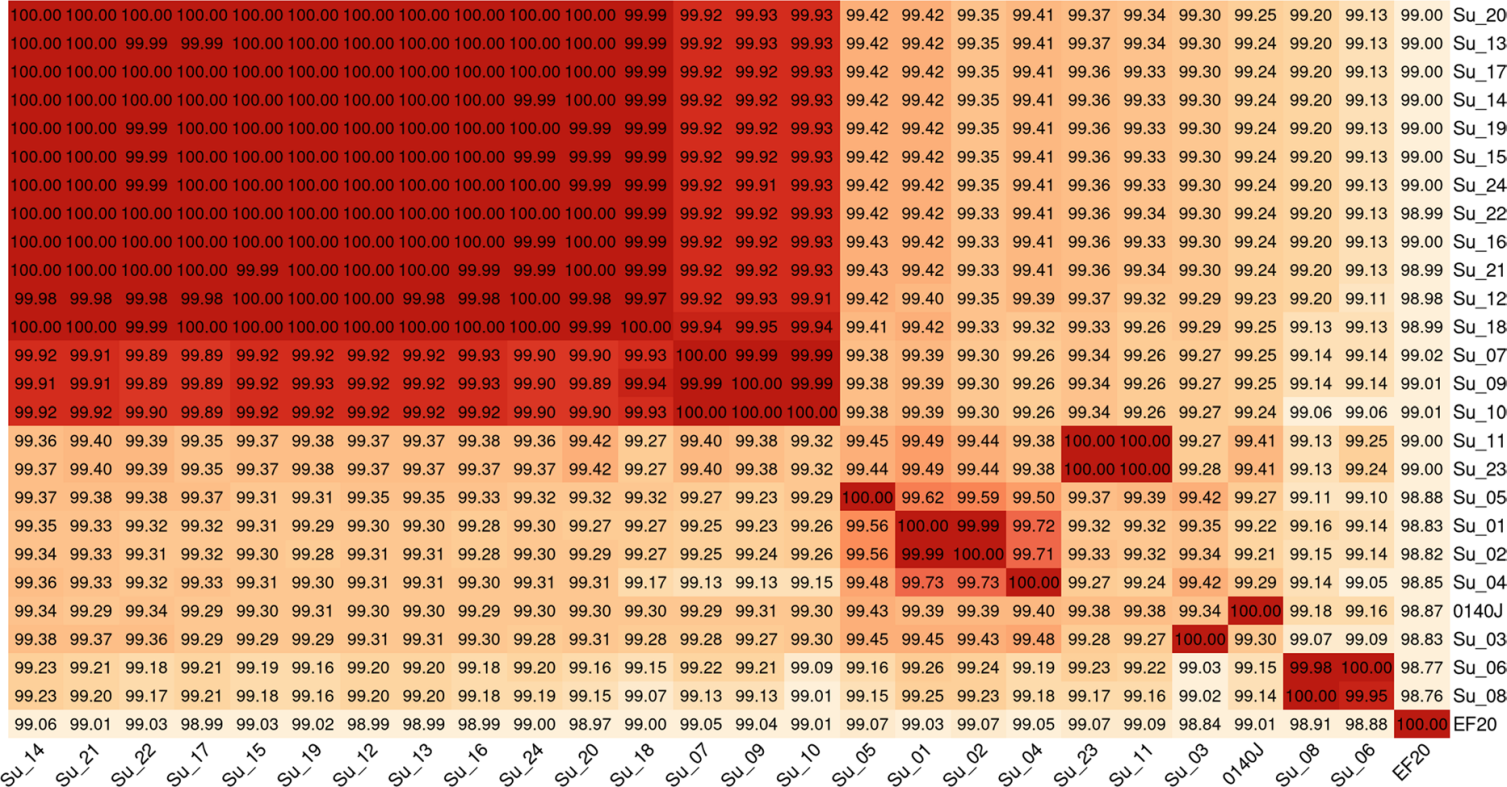
Overview of the average nucleotide match between genomes. The isolates were grouped according to their match. Within each box, the identity was given in percent. Darker heat map colours indicate higher relatedness. Results were obtained using EDGAR 3.0 based on a BLASTN comparison of the genome sequences

To get a first impression of the intra-species similarity between isolates, an average nucleotide identity (ANI) matrix was created (Fig. 4). The ANI between all isolates was within 98.37 % and 100 %. Isolates Su-01 and Su-02 gave a 100 % match to each other, as did isolates Su-11 and Su-23. In addition, a group of isolates that also completely matched in their ANI could be identified within group C (Su-14 to Su-17, Su-19 to Su-22, and Su-24).

### Multilocus sequence typing

Four of the isolates, as well as the reference isolates, could be assigned to previously known sequence types (ST) namely ST931 (n = 2), ST964 (n = 2), ST55 (n = 1) and ST1 (n = 1). All other isolates, contained allele variants that have not yet been described. In detail, seventeen of the isolates contained new allele variations of the *tdk* gene locus, which were registered as allele numbers 122, 123 and 124 in the *S. uberis* MLST database (pubmlst.org/suberis). With registration of the new locus variants, all remaining isolates could be assigned to new sequence types, namely ST1373 (n = 12), ST1374 (n = 3), ST1375 (n = 2), ST1377 (n = 1), ST1378 (n = 1) and ST1379 (= 1). Overall, we detected four different STs among isolates from farm A (ST1375, ST1377, ST1378, ST1379), two STs in farm B (ST931 and ST1374), and two STs in farm C (ST964 and ST1373) (Table 4). Thus, 12 of the 14 isolates (85.7%) from group C showed the same MLST type. The novel STs ST1373 and ST1374 belonged to clonal complex CC5, with possible founder ST1065. All other STs were double locus variants (DLV).

**Table 4:** MLST results for 24 isolates plus the strains 0140J and EF20

Isolate	Disease status	Allele							ST	CC
		*arcC*	*ddl*	*gki*	*recP*	*tdk*	*tpi*	*yqiL*		
*Group A*										
Su-01	C	1	37	4	2	62	1	3	**1375**	–
Su-02	C	1	37	4	2	62	1	3	**1375**	–
Su-03	C	1	1	4	2	**123**	1	3	**1377**	–
Su-04	C	2	33	4	2	**124**	1	3	**1378**	–
Su-05	C	1	37	5	2	62	1	3	**1379**	–
*Group B*										
Su-06	C	2	1	10	3	2	3	3	931	–
Su-07	C	2	1	5	1	**122**	1	3	**1374**	5
Su-08	C	2	1	10	3	2	3	3	931	–
Su-09	C	2	1	5	1	**122**	1	3	**1374**	5
Su-10	C	2	1	5	1	**122**	1	3	**1374**	5
*Group C*										
Su-11	C*	2	37	5	1	97	1	3	964	–
Su-12	C*	2	1	65	1	**122**	1	3	**1373**	5
Su-13	C*	2	1	65	1	**122**	1	3	**1373**	5
Su-14	C*	2	1	65	1	**122**	1	3	**1373**	5
Su-15	C*	2	1	65	1	**122**	1	3	**1373**	5
Su-16	C*	2	1	65	1	**122**	1	3	**1373**	5
Su-17	C*	2	1	65	1	**122**	1	3	**1373**	5
Su-18	C*	2	1	65	1	**122**	1	3	**1373**	5
Su-19	C*	2	1	65	1	**122**	1	3	**1373**	5
Su-20	C*	2	1	65	1	**122**	1	3	**1373**	5
Su-21	C*	2	1	65	1	**122**	1	3	**1373**	5
Su-22	C*	2	1	65	1	**122**	1	3	**1373**	5
Su-23	C*	2	37	5	1	97	1	3	964	–
Su-24	C*	2	1	65	1	**122**	1	3	**1373**	5
*Reference strains*										
0140J	–	1	1	1	1	1	1	1	1	5
EF20	–	15	1	4	3	13	4	3	55	–

In bold are the allele variants and resulting STs that were novel in this study. Isolates marked with asterisks (*), showed the particularly severe disease status

### Virulence factors

**Fig. 5:**
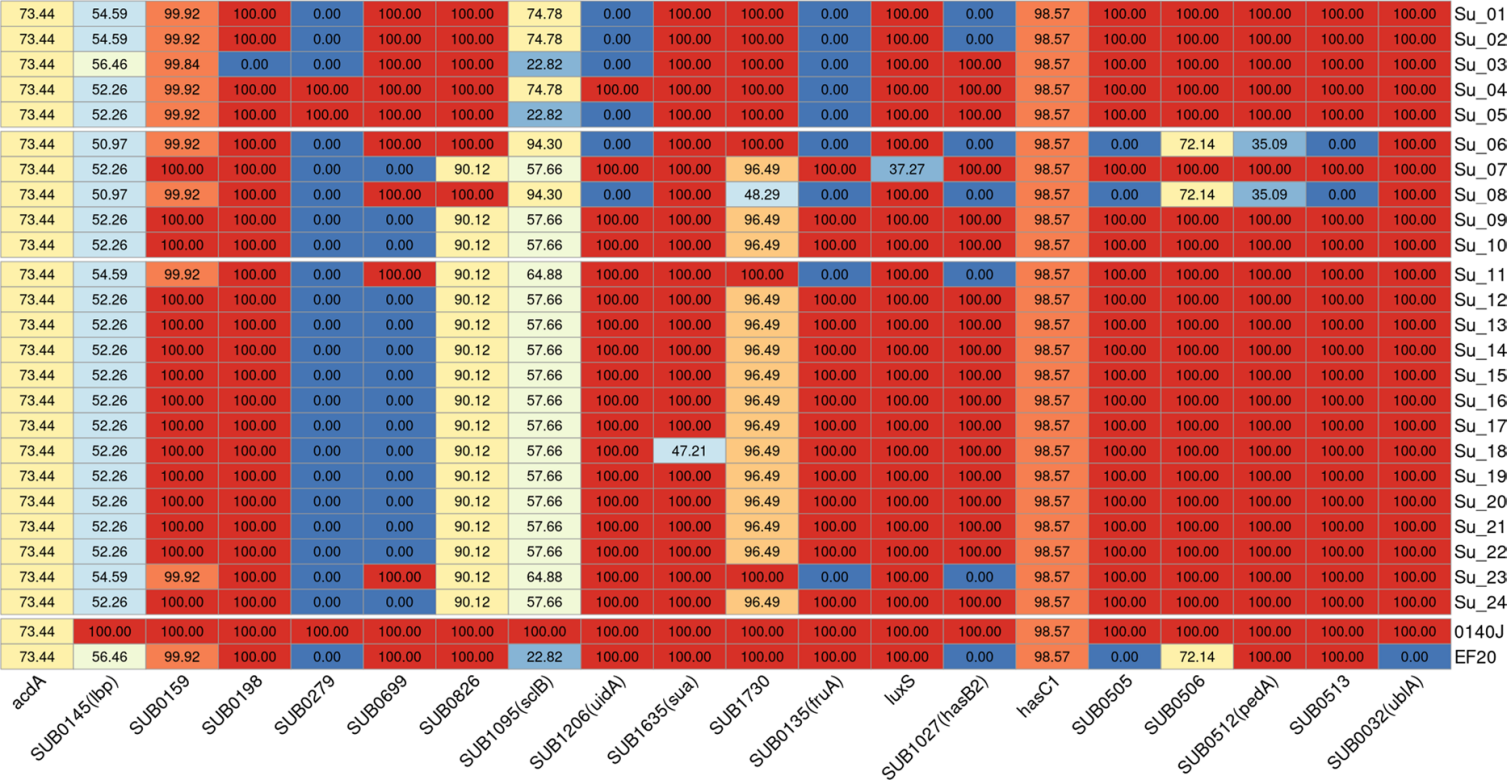
All virulence genes that were not found in all isolates with a gene coverage of 100 % are listed. The gene-coverage in percent is indicated in the boxes. The heatmap was created with R-Studio from the results generated with ABRicate

In total, 66 putative virulence genes were found. Forty-eight of these occurred in all isolates with 100 % gene coverage. All genes examined were also detected in strain 0140J and strain EF20 likewise contained all but four of these genes. The genes for zinc binding protein (*acdA*)*,* lactoferin binding protein (locus tag in 0140J genome: *SUB0145*; gene name: *lbp*)*,* conserved hypothetical protein (*SUB0159*)*,* putative surface-anchored subtulase family protein (*SUB0826*)*,* collagen-like surface-anchored protein (*SUB1095*; *sclB*)*,* S-ribosylhomocysteinase (*luxS*) and the putative bacteriocins locus tags/genes *SUB0506* and *pedA* were detected in all isolates, but not with complete gene coverage (Fig. 5). Virulence properties in group C isolates were highly homologue and the majority of all isolates matched identical virulence genes. The isolate Su-18 showed a lower coverage for the locus tag *SUB1635.* The isolates Su-11 and Su-23 lacked the capsule gen *hasB2* (*SUB1027*) and the gene for the putative fructan beta-fructosidase precursor (*SUB0135; fruA*) but carried the gene for the glycosyl transferase (*SUB0699*)*.* A total list of all genes with accession numbers and references can be found in Suppl. Table 2.

### Antimicrobial resistance genes

In the search for putative AMR genes, those for the penicillin-binding-proteins (*pbp1a, pbp1b, pbp2a* and *pbp2x*) were found in all isolates. In the sequence of *pbp1b* an amino acid substitution G_769_S occurred in all isolates, with the exception of Su-06 and Su-08, as well as the strain EF20. In addition, the isolates Su-01 to Su-04 were found to contain the substitutions P_746_Q and N_117_K. In the gene *pbp2a,* the substitution I_330_V was determined in all isolates, whereas the substitution Q_321_H could only be detected in isolates Su-11 and Su-23. The isolates Su-02, Su-03, Su-05 as well as the strain EF20 contained an amino acid substitution K_43_E. Whereas T_36_A was substituted in isolates Su-06 and Su-08. Gene *pbp2x* showed substitutions E_381_K, Q_554_E, and G_600_E in all isolates except Su-11 and Su-23. All isolates were tested sensitive to typical *β*-lactam antibiotics in vitro (Table 2). Further putative resistance genes included hits in Su-06 and Su-08 containing the gene *lnuC*, whereas Su-01, Su-02, Su-04 and Su-05 carried the gene *lnuD,* both of which can lead to resistance to lincosamides. In Su-01 and Su-02, the gene *tetS*, coding for tetracycline resistance, was also confirmed. Furthermore, the gene *qacH* (*SUB0162*), which may leads to resistance to quaternary ammonium compounds, was found in all isolates with a coverage ranging from at least 50 % to as high as 100 % in the isolates Su-06, Su-08, Su-11 and Su-23.

### Phages

**Fig. 6:**
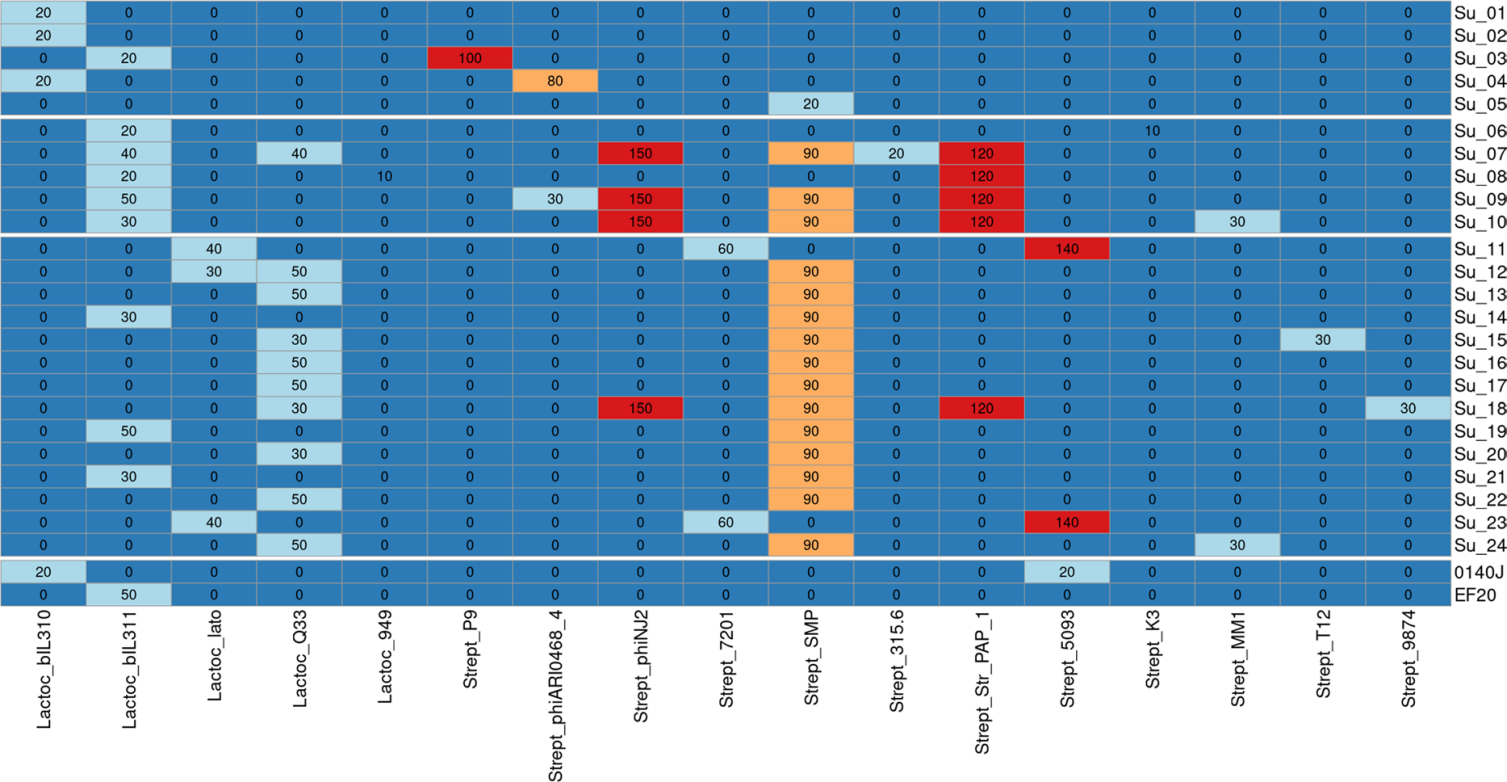
All prophage sequences found are listed. The score (< 70: incomplete; 70–90: questionable; > 90: intact) for each sequence is indicated in the boxes. The heatmap was created with R-Studio from the results generated with PHASTER

A total of 12 different phages were found in all isolates, with the most commonly detected phages being *Streptococcus* phage SMP and *Lactococcus* phage bIL311 (Fig. 6; detailed summary in Suppl. Table 3). The *Streptococcus* phage phiNJ2 (Strept_phiNJ2) was most abundant as an intact region. Overall eight of the 24 isolates had intact prophage regions.

## Discussion

The aim of this study was to identify genetic differences and similarities between 24 *Streptococcus uberis* isolates that proved responsible for highly different disease outcomes. Of particular interest was the question, whether there were clearly identifiable reasons as to why the isolates from farm C led to such a severe infection. Recent studies have found a smaller genome size in virulent strains of the closely related bacterial pathogen *S. suis* (Murray et al. 2021) and other studies also hypothesised this for *S. uberis* (Hossain et al. 2015). Despite the evidence for significant differences in the genome size of our isolates, there was no linkage between small genome size and potentially epidemic isolates, associated with higher virulence and a severe course of infection, or higher genome size and isolates that caused sporadic subclinical or clinical mastitis. Although group B isolates displayed significantly higher genome sizes compared to the other two groups, group A had the smallest genome size on average (as measured by total assembled contig size). However, it should be noted that group A and B are prone to relatively small sample sizes and therefore, the results are not necessarily exhaustive. Although the genomes examined in this study have a lower mean genome size than those currently found in the NCBI database, it is not clear whether there is a correlation between genome size and virulence, as not all genomes in the database are clearly labelled as clinical or subclinical isolates. Further studies are needed to fully clarify a potential correlation. Due to the similarity of the isolates from group C, the assumption of a clonal outbreak was reasonable to suspect. The phylogenetic analysis revealed a most closely related population of all but two isolates from group C, further suggesting a clonal relationship in these isolates that represented the vast majority of the outbreak isolates in this herd. However, two isolates (Su-11, Su-23) from the same group clustered closer together with the strain 0140J, thereby supporting the general opinion of multiple, genetically non-related *S. uberis* within one herd. Interestingly, isolates Su-07, Su-09 and Su-10 from the randomly chosen farm B seemed to be next closely related to the clonal group of the isolates from farm C. The stability of the pan and core genomes within group C confirms the concept of a clonal outbreak and is consistent with the results of the phylogenetic tree.

Future studies should elucidate, whether the supposed clonal outbreak isolates from this study indeed represent a dominant genotype among other *S. uberis* strains that is linked with increased virulence and possibly with further geographic distribution.

With respect to MLST, 17 out of 24 isolates showed novel alleles, reflecting the high diversity in *S. uberis* as has already been mentioned previously in a similar study carried out with dairy cows from Australia (Vezina et al. 2021). Interestingly, the gene for the thymidine kinase (*tdk*) shows the most new variants here, also proven by the fact that the gene locus *tdk* is the most variable allele variant within the pubMLST database with 124 variants, whereas only 37 to 85 variants are stored for the other gene loci. This could be taken as a reason to believe that this gene is not as stable as other housekeeping genes.

The similarity of the allele profiles in group C as well as their phylogenetic position in the core genome tree confirms once again that most of the isolates are clones of the same type, which fosters to speculate that the spreading event inside the herd was caused by infected cows rather than from the environment. Nevertheless. it should not be disregarded, that this type could prevail as a dominant type in the environment and thus leads to predominant udder infections. Previous findings on this pathogen have also suggested that 50–100 % of all animals are infected with the same or a very similar strain in some herds (Zadoks et al. 2003). Despite the smaller sample size, this study also shows a great diversity of STs, mostly indicating a heterogeneous than contagious spread of *S. uberis* (Käppeli et al. 2019). However, the almost exclusive occurrence of ST1373 within group C as a new ST, could point to a contagious event with ST1373 as a ST possibly strongly associated with virulence. In addition, the two STs ST1373 and ST1374 belong to the clonal cluster CC5, which is most commonly associated with clinical mastitis (Zadoks et al. 2011).

Considering the equipment with virulence genes, all strains from this study principally appear to have a considerable virulence potential. More than half of all detected putative virulence factors occurred in all 24 isolates with complete gene coverage and almost complete sequence identity, indicating strong conservation of these sequences. As a significant finding contrary to our hypothesis, this would suggest that no significant differences do exist in the repertoire of known virulence genes that predispose isolates for causing severe and possibly epidemic or just local infections. This is also supported by the fact that, as already stated, the three isolates from farm B clustered closely together with all isolates from farm C. However, another possible explanation could indicate that epidemic isolates have a better adaptation to the udder tissue or that the animals were immunocompromised by pretreatments. In line with the traditional concept that *S. uberis* represents a pathogen whose pathogenic potential is highly dependent on environmental factors and host immune system, our results suggest that the fulminant disease progression in farm C was probably more likely being influenced by such factors than by a specific set of genes in the outbreak strain. No systematic data were available for the general health situation in farms A, B and C, but the accumulation of *S. uberis* mastitis in all these herds suggests an a priori sub-optimal immune status that is most often related to deficiencies in animal keeping, nutrition, co-infections or other health issues (Günther et al. 2016).

It is rather difficult to make a clear statement as to whether certain phages contribute to increased virulence here, because intact regions could not be found in any of the used reference strains. In addition, most intact phage regions were found within group B, consisting of isolates from local, rather mild infections. Thus, this will be the reason why in this group the mean genome size is higher than in the other groups. In any case, the diversity of prophage regions in this group may be indicative of phage adaptation to new hosts.

The size of the core genome of all 24 isolates is slightly higher than the results in other studies, whereas the size of the pan genome is smaller (Lang et al. 2009; Hossain et al. 2015; Vezina et al. 2021). This further suggests that *S. uberis* appears to be a genetically highly diverse organism. Six of the 24 isolates carried either *lnuC* or *lnuD* as putative antimicrobial resistance genes. While *lnuD* has already been found in *S. uberis* isolates from cases of mastitis, *lnuC* has so far mostly been reported in *S. agalactiae,* which could be an indication for lateral gene transfer according to (Vezina et al. 2021). The three substitutions found in the gene *pbp2x* could lead to increased resistance to oxacillin, according to the results of McDougall et al. 2020. This is a cause for concern, as *β*-lactam antibiotics, particularly penicillin G are first choice antimicrobials to treat *S. uberis* infections and are used during infection prophylaxis in dry cows, both of which could promote further mutations and resistance. The substitutions found in the other genes for penicillin binding proteins (*pbp1b*, *pbp2a*) have not yet been linked to *β*-lactam resistance. Nevertheless, since the substitutions are present in the majority of all isolates, i.e. seem to be evolutionary stable, it should not be completely ruled out that they could contribute to increased resistance. However, all isolates were tested sensitive to penicillin and oxacillin in vitro, which means that functional resistance cannot be confirmed here.

The gene *qacH* (*SUB0162*) has been described in previous studies with 41 % identity to the gene QACH_STASA in *Staphylococcus saprophyticus,* which codes for a quaternary ammonium compound-resistance protein (Ward et al. 2009). Disinfectants containing such compounds are often routinely used as part of the daily milking procedure and could imply a certain selective pressure towards these substances. The fact that all isolates as well as both reference strains carried this gene should raise concern and warrant in vitro susceptibility testing as well as considering the prudent use and regularly change of other disinfectants in milking hygiene.

## Conclusion

Comparison of all isolates revealed no clear set of genes responsible for enhanced virulence. Furthermore, no indubitable differences in overall gene content were found. However, it should be noted that most isolates within group C are highly similar, thus a spreading event from cow to cow and a common course for the specific disease progression is very likely. Furthermore, our study has also shown a highly conserved set of virulence genes. This should open new avenues for the search of potential vaccine targets. It also remains to be observed in more detail whether strains with fewer or no virulence genes are also responsible for mastitis cases or severe infections. To gain further clarity on the pathogenicity of *S. uberis*, one should include also host genomes in addition to bacterial genomes as well as the environmental circumstances. Such further metagenomic studies may reveal factors that can be used to better control the spread of *S. uberis* and thus reduce the risk of mastitis and the high use of antibiotics.

## Supplementary Information

Below is the link to the electronic supplementary material.

Supplementary file1 (DOCX 29 KB)

## Data Availability

All genomes sequenced in this study are available at https://pubmlst.org; Ids 2255–2287 and at NCBI under the Bioproject: PRJNA795889. The datasets generated during and/or analysed during the current study are available from the corresponding author on reasonable request.
