# Supplementary material for: A dominant clonal lineage of Streptococcus uberis in cattle in Germany
Source: Antonie Van Leeuwenhoek. 2022 Apr 30;115(7):857–70. doi: 10.1007/s10482-022-01740-w (PMC9206625; doi:10.1007/s10482-022-01740-w)
Supplement: Supplementary file 1 — Supplementary file1 (DOCX 29 KB) [file 10482_2022_1740_MOESM1_ESM.docx]

**A dominant clonal lineage of *Streptococcus uberis* in cattle in Germany**

Linda Fenske^1,2*^, Irene Noll^3^, Jochen Blom^2^, Christa Ewers^4^, Torsten Semmler^5^, Ahmad Fawzy^6,1^, Tobias Eisenberg^1,4^

^1^ Hessian State Laboratory, Department of Veterinary Medicine, Giessen, Germany

^2^ Bioinformatics and Systems Biology, Justus-Liebig-University, Giessen, Germany

^3^ Regional Council of Gießen, Wetzlar, Germany

^4^ Institute of Hygiene and Infectious Diseases of Animals, Justus Liebig University, Giessen, Germany

^5^ NG 1 Microbial Genomics, Robert Koch Institute (RKI), Berlin, Germany

^6^ Cairo University, Faculty of Veterinary Medicine, Department of Medicine and Infectious Diseases, Giza, Egypt

*corresponding author, mailing address: M.Sc. Linda Fenske, Bioinformatics and Systems Biology,
Justus Liebig University, Giessen, Germany, linda.lenske@computational.bio.uni-giessen.de

**Suppl. Table 1** More detailed information on the breed, age and origin of the cows from which the samples were taken

| **Isolate** | **Country** | **Federal State** | **Year** | **Source** | **Sample** | **Breed** | **Age [years]** |
| --- | --- | --- | --- | --- | --- | --- | --- |
| **Group A** | | | | | | | |
| Su-01 | Germany | Rhineland-Palatinate | 2019 | cow (udder) | milk | German Black Pied cattle | 11 |
| Su-02 | Germany | Rhineland-Palatinate | 2019 | cow (udder) | milk | German Black Pied cattle | 7 |
| Su-03 | Germany | Rhineland-Palatinate | 2019 | cow (udder) | milk | German Black Pied cattle | 5 |
| Su-04 | Germany | Rhineland-Palatinate | 2019 | cow (udder) | milk | German Black Pied cattle | 8 |
| Su-05 | Germany | Rhineland-Palatinate | 2019 | cow (udder) | milk | German Black Pied cattle | 5 |
| **Group B** | | | | | | | |
| Su-06 | Germany | Hesse | 2019 | cow (udder) | milk | German Black Pied cattle | 5 |
| Su-07 | Germany | Hesse | 2019 | cow (udder) | milk | German Black Pied cattle | 2 |
| Su-08 | Germany | Hesse | 2019 | cow (udder) | milk | German Black Pied cattle | 4 |
| Su-09 | Germany | Hesse | 2019 | cow (udder) | milk | German Black Pied cattle | 5 |
| Su-10 | Germany | Hesse | 2019 | cow (udder) | milk | German Black Pied cattle | 2 |
| **Group C** | | | | | | | |
| Su-11 | Germany | Hesse | 2016 | cow (udder) | milk | German Red Pied cattle | 6 |
| Su-12 | Germany | Hesse | 2016 | cow (udder) | milk | German Red Pied cattle | 6 |
| Su-13 | Germany | Hesse | 2016 | cow (udder) | milk | German Black Pied cattle | 7 |
| Su-14 | Germany | Hesse | 2016 | cow (udder) | milk | German Black Pied cattle | 7 |
| Su-15 | Germany | Hesse | 2016 | cow (udder) | milk | German Black Pied cattle | 6 |
| Su-16 | Germany | Hesse | 2016 | cow (udder) | milk | German Black Pied cattle | 6 |
| Su-17 | Germany | Hesse | 2016 | cow (udder) | milk | German Black Pied cattle | 6 |
| Su-18 | Germany | Hesse | 2016 | cow (udder) | milk | German Black Pied cattle | 6 |
| Su-19 | Germany | Hesse | 2016 | cow (udder) | milk | German Black Pied cattle | 9 |
| Su-20 | Germany | Hesse | 2016 | cow (udder) | milk | German Black Pied cattle | 7 |
| Su-21 | Germany | Hesse | 2016 | cow (udder) | milk | German Black Pied cattle | 9 |
| Su-22 | Germany | Hesse | 2016 | cow (udder) | milk | German Black Pied cattle | 7 |
| Su-23 | Germany | Hesse | 2016 | cow (udder) | milk | German Black Pied cattle | 6 |
| Su-24 | Germany | Hesse | 2016 | cow (udder) | milk | German Black Pied cattle | 6 |

**Suppl. Table 2** All genes found with ABRicate are listed. Genes found in all 24 isolates with 100 % gene coverage are highlighted in light gray. The specific uniprot acession numbers are given in parentheses at the end of the function. In addition, reference is made to studies which have also analysed these genes.

| **Locus tag in the genome of 0140J  (gene name)** | **Function** | **Reference** |
| --- | --- | --- |
| **I) Putative virulence genes and anchor proteins** | | |
| *gapC* | Glyceraldehyde-3-phosphate dehydrogenase (AF421900) | Reinoso et al. 2011 Ballas et al. 2020 |
| *acdA* | Zinc-binding protein (AAL00777.1) | Fessia et al. 2019 Ballas et al. 2020 |
| *SUB0135 (fruA)* | Putative fructan beta-fructosidase precursor (CAR40555.1) | Leigh et al. 2010 Hossain et al. 2015 Crowley et al. 2011 Collado et al. 2016 |
| *SUB0144 (vru)* | Putative Mga-like regulatory protein (CAR40575.1) | Abureema et al. 2019 |
| *SUB0145 (lbp)* | Lactoferrin binding protein (CAR40577.1) | Reinoso et al. 2011 Fessia et al. 2019 Leigh et al. 2010 Hossain et al. 2015 Collado et al. 2016 Ward et al. 2009 |
| *SUB0241* | Putative surface-anchored 2’3-cyclic-nucleotide 2’-phosphodiesterase (CAR40749.1) | Leigh et al. 2010 Hossain et al. 2015 Ward et al. 2009 |
| *SUB0826* | Putative surface-anchored subtilase family protein (CAR41854.1) | Leigh et al. 2010 Hossain et al. 2015 |
| *SUB0881 (SrtA)* | Sortase A (CAR41958.1) | Hossain et al. 2015 Collado et al. 2016 |
| *SUB0888* | Putative surface-anchored protein (CAR41974.1) | Leigh et al. 2010 Hossain et al. 2015 Ward et al. 2009 |
| *SUB0207* | Putative surface-anchored protein (CAR40684.1) | Leigh et al. 2010 Hossain et al. 2015 Ward et al. 2009 |
| *SUB1730* | Putative surface-anchored protein (CAR43675.1) | Leigh et al. 2010 Hossain et al. 2015 Ward et al. 2009 |
| *SUB1095 (sclB)* | Collagen-like surface protein (CAR42417.1) | Leigh et al. 2010 Hossain et al. 2015 Collado et al. 2016 Ward et al. 2009 |
| *SUB1111 (fbpS)* | Fibronectin-binding protein (CAR42461.1) | Fessia et al. 2019 Ballas et al. 2020 Hossain et al. 2015 Collado et al. 2016 |
| *SUB1154 (C5a)* | C5a peptidase precursor (CAR42550.1) | Leigh et al. 2010 Hossain et al. 2015 Collado et al. 2016 |
| *SUB1273* | Hemolysin-like protein (CAR42774.1) | Hossain et al. 2015 Collado et al. 2016 |
| *SUB1370* | Putative zinc-carboxypeptidase (CAR42963.1) | Leigh et al. 2010 Hossain et al. 2015 |
| *SUB1785 (pauA)* | Streptokinase precursor (CAR43782.1) | Reinoso et al. 2011 Ballas et al. 2020 Hossain et al. 2015 Ward et al. 2009 |
| *SUB1635 (sua)* | Streptococcus uberis adhesion molecule (CAR43489.1) | Reinoso et al. 2011 Fessia et al. 2019 Ballas et al. 2020 Hossain et al. 2015 Ward et al. 2009 |
| *SUB0159* | Conserved Hypothetical Protein (CAR40602.1) | Crowley et al. 2011 |
| *oppF* | Partial oligopeptide permease-like protein (ADZ16846.1) | Reinoso et al. 2011 |
| **II) Putative genes for biofilm production** | | |
| *SUB0330 (fba)* | Fructose-biphosphate aldolase (CAR40904.1) | Collado et al. 2016 |
| *SUB0063 (adhE)* | Alcohol-acetaldehyde dehydrogenase 2 (CAR40428.1) | Crowley et al. 2011 |
| *SUB0161 (fsaA)* | Fructose-6-phosphate adolase ( CAR40606.1) | Crowley et al. 2011 Collado et al. 2016 |
| *SUB0198* | Putative beta-glucosidase (CAR40665.1) | Salomäki et al. 2015 Ward et al. 2009 |
| *SUB0232* | Hypothetical protein (CAR40732.1) | Salomäki et al. 2015 |
| *SUB0235* | Transcriptional regulator - LacI family (CAR40732.1) | Crowley et al. 2011 Collado et al. 2016 |
| *SUB0279* | Type I restriction modification system DNA methylase (CAR40812.1) | Salomäki et al. 2015 Crowley et al. 2011 |
| *SUB0429 (clpP)* | ATP-dependent Clp protease proteolytic subunit (CAR41093.1) | Salomäki et al. 2015 |
| *SUB0440* | Hypothetical protein (CAR41108.1) | Salomäki et al. 2015 |
| *SUB0699* | Glycosyl transferase ( CAR41604.1) | Salomäki et al. 2015 |
| *SUB0719 (gor)* | Glutathione reductase (CAR41636.1) | Crowley et al. 2011 |
| *SUB0750* | Putative fructose-specific phosphotransferase system (CAR41692.1) | Crowley et al. 2011 Collado et al. 2016 |
| *SUB0837* | 6-phospho-beta-glucosidase 4 (CAR41871.1) | Crowley et al. 2011 Collado et al. 2016 Ward et al. 2009 |
| *SUB0877 (nox)* | Glycosyl transferase (CAR41944.1) | Salomäki et al. 2015 |
| *SUB0944 (coaC)* | Phosphopantothenoylcysteine decarboxylase (AR40944.1) | Salomäki et al. 2015 |
| *SUB1003* | GntR family regulatory protein (CAR42240.1) | Crowley et al. 2011 |
| *SUB1134 (miaA)* | tRNA delta(2)-isopentenylpyrophosphate transferase (CAR42507.1) | Salomäki et al. 2015 |
| *SUB1141 (malM)* | 4-alpha-glucanotransferase (CAR42521.1) | Crowley et al. 2011 |
| *SUB1152* | Putative Glutamine ABC transporter (CAR42546.1) | Crowley et al. 2011  Collado et al. 2016 |
| *SUB1206 (uidA)* | Beta-glucuronidase (CAR42644.1) | Salomäki et al. 2015 |
| *SUB1212* | Lipoprotein/Internalin homologue (CAR42655.1) | Crowley et al. 2011 Collado et al. 2016 |
| *SUB1382 (liaR)* | Response regulator protein (CAR42990.1) | Salomäki et al. 2015 |
| *SUB1499 (acpP)* | Acyl carrier protein ( CAR43216.1) | Crowley et al. 2011 |
| *SUB1794 (hisS)* | Histidyl-tRNA synthetase (CAR43804.1) | Crowley et al. 2011 |
| *SUB1810* | NUDIX hydrolyase (CAR43838.1) | Salomäki et al. 2015 |
| *comEA* | Putative competence protein (CAR42718.1) | Moore, 2009 |
| *comEC* | Putative competence protein (CAR42715.1) | Moore, 2009 |
| *comX* | Putative competence-specific global transcription modulator (CAR40944.1) | Moore, 2009 |
| *luxS* | S-ribosylhomocysteinase (CAR43029.1) | Moore, 2009 |
| *SUB1118* | Conserved hypothetical protein (CAR42474.1) | Crowley et al. 2011 |
| *SUB0884 (lmb)* | Laminin binding protein (CAR41964.1) | Fessia et al. 2019 |
| *SUB0405 (scpA)* | Segretation and condensation protein A (CAR41047.1) | Fessia et al. 2019 |
| **III) Capsule production** | | |
| *SUB1697 (hasA)* | Hyaluron-Synthase (CAR43611.1) | Reinoso et al. 2011 Ward et al. 2001 Ballas et al. 2020 |
| *SUB1696 (hasB)* | UDP-glucose 6-dehydrogenase 1 (CAR43609.1) | Reinoso et al. 2011 Ward et al. 2001 Ballas et al. 2020 |
| *SUB1027 (hasB2)* | UDP-glucose 6-dehydrogenase 2 (CAR42292.1) | Ballas et al. 2020 Ward et al. 2009 |
| *hasC1* | UTP-glucose-1-phosphate uridylyltransferase (WP010922799) | Reinoso et al. 2011 Ward et al. 2001 Ballas et al. 2020 |
| *SUB1691 (gpsA)* | Glycerol-3-phosphate dehydrogenase (CAR43601.1) | Ward et al. 2009 |
| **IV) Bacteriocine** | | |
| *SUB0505* | Putative Bacteriocin ( CAR41231.1) | Hossain et al. 2015 Ward et al. 2009 |
| *SUB0506* | Putative Bacteriocin ([CAR41232.1](https://www.ebi.ac.uk/ena/data/view/CAR41232) ) | Hossain et al. 2015 Ward et al. 2009 |
| *SUB0509* | Putative Bacteriocin (CAR41236.1) | Hossain et al. 2015 Ward et al. 2009 |
| *SUB0510* | Putative Bacteriocin immunity protein (CAR41238.1) | Ward et al. 2009 |
| *SUB0512 (pedA)* | Putative Bacteriocin (CAR41244.1) | Hossain et al. 2015 |
| *SUB0513* | Putative Bacteriocin immunity protein (CAR41245.1) | Ward et al. 2009 |
| *SUB0516* | Putative Bacteriocin immunity protein (CAR41255.1) | Ward et al. 2009 |
| *SUB0032 (ublA)* | Putative Bacteriocin (Uberolysin) (CAR40379.1) | Wirawan et al. 2007 Ward et al. 2009 |
| **V) Putative antimicrobial resistance genes** | | |
| *lnuC* | Linosamide nucleotidyltransferase (AY928180) | Haenni et al. 2011 Vezina et al. 2021 |
| *lnuD* | Linosamide nucleotidyltransferase ( EF452177) | Vezina et al. 2021 |
| *tetS* | Tetracycline-resistant ribosomal protection protein (L09756) | Vélez et al. 2017 |
| *qacH (SUB0162)* | Quaternary ammonium compound resistance protein (CAR40609.1) | Ward et al. 2009 |

**Suppl. Table 3** Distribution of prophage regions of 24 isolates plus the references 0140J and EF20. Results generated with PHASTER. Score: < 70: incomplete; 70-90: questionable; > 90: intact. Acession numbers are given parantheses

| **Isolate** | **Putative phage** | **Status** | **Score** | **Length (kb)** | **GC%** |
| --- | --- | --- | --- | --- | --- |
| **Group A** | | | | | |
| Su-01 | Lactoc_bIL310 (NC_002669.1) | incomplete | 20 | 15.5 | 33.91 |
| Su-02 | Lactoc_bIL310 (NC_002669.1) | incomplete | 20 | 15.4 | 33.95 |
| Su-03 | Strep_P9 (DQ864624.1) | intact | 100 | 41.0 | 36.10 |
|  | Lactoc_bIL311(NC_002670.1) | incomplete | 20 | 15.3 | 34.10 |
| Su-04 | Strept_phiARI0468_4 (NC_031915) | intact | 80 | 57.6 | 36.36 |
|  | Lactoc_bIL310 (NC_002669.1) | incomplete | 20 | 15.5 | 33.91 |
| Su-05 | Strept_SMP (EF116926.2) | incomplete | 20 | 13.8 | 34.18 |
| **Group B** | | | | | |
| Su-06 | Strept_K13 (NC_24357.1) | incomplete | 10 | 28.7 | 35.15 |
|  | Lactoc_bIL311 (NC_002670.1) | incomplete | 20 | 16.2 | 34.72 |
| Su-07 | Lactoc_Q33 (NC_049809) | incomplete | 40 | 28.1 | 34.70 |
|  | Strept_SMP (EF116926.2) | questionable | 90 | 38.4 | 35.13 |
|  | Strept_Str_PAP_1 (NC_028666.1) | intact | 120 | 39.7 | 38.70 |
|  | Strept_phiNJ2 (NC_019418) | intact | 150 | 43.1 | 36.35 |
| Su-08 | Lactoc_949 (NC_015263.1) | incomplete | 10 | 28.7 | 35.15 |
|  | Lactoc_bIL311 (NC_002670.1) | incomplete | 20 | 16.3 | 34.68 |
|  | Strept_Str_PAP_1 (NC_28666.1) | intact | 120 | 39.4 | 36.67 |
| Su-09 | Lactoc_bIL311 (NC_002670.1) | incomplete | 50 | 32.9 | 34.91 |
|  | Strept_SpSL1 (NC_027396) | incomplete | 30 | 20.5 | 36.99 |
|  | Strept_phiNJ2 (NC_019418) | intact | 150 | 45.2 | 36.32 |
|  | Strept_SMP (EF116926.2) | questionable | 90 | 38.4 | 35.13 |
|  | Strept_Str_PAP_1 (NC_28666.1) | intact | 120 | 38.3 | 38.74 |
| Su-10 | Strept_MM1 (NC_003050.2) | incomplete | 30 | 19.2 | 37.14 |
|  | Strept_phiNJ2 (NC_019418) | intact | 150 | 45.2 | 36.31 |
|  | Strept_SMP (EF116926.2) | questionable | 90 | 38.4 | 35.13 |
|  | Strept_Str_PAP_1 (NC_28666.1) | intact | 120 | 39.6 | 38.73 |
|  | Lactoc_bIL311 (NC_002670.1) | incomplete | 30 | 28.5 | 35.47 |
| **Group C** | | | | | |
| Su-11 | Lactoc_lato (NC_004746) | incomplete | 40 | 35.7 | 34.56 |
|  | Strept_7201 (NC_002185) | incomplete | 60 | 43.5 | 34.69 |
|  | Strept_5093 (NC_012753) | intact | 140 | 46.6 | 36.05 |
| Su-12 | Lactoc_Q33 (NC_049809) | incomplete | 50 | 32.9 | 34.91 |
|  | Lactoc_lato (NC_004746) | incomplete | 30 | 20.5 | 37.00 |
|  | Strept_SMP (EF116926.2) | questionable | 90 | 38.8 | 35.12 |
| Su-13 | Lactoc_Q33 (NC_049809) | incomplete | 50 | 32.9 | 34.91 |
|  | Strept_SMP (EF116926.2) | questionable | 90 | 38.8 | 35.12 |
| Su-14 | Lactoc_bIL311 (NC_002670.1) | incomplete | 30 | 28.5 | 35.46 |
|  | Strept_SMP (EF116926.2) | questionable | 90 | 38.8 | 35.12 |
| Su-15 | Strept_T12 (NC_028700) | incomplete | 30 | 19.2 | 37.14 |
|  | Lactoc_Q33 (NC_049809) | incomplete | 30 | 28.5 | 35.46 |
|  | Strept_SMP (EF116926.2) | questionable | 90 | 38.8 | 35.12 |
| Su-16 | Lactoc_Q33 (NC_049809) | incomplete | 50 | 32.9 | 34.91 |
|  | Strept_SMP (EF116926.2) | questionable | 90 | 38.8 | 35.12 |
| Su-17 | Lactoc_Q33 (NC_049809) | incomplete | 40 | 32.9 | 34.91 |
|  | Strept_SMP (EF116926.2) | questionable | 90 | 38.8 | 35.12 |
| Su-18 | Strept_9874 (NC_031023) | incomplete | 30 | 19.2 | 37.14 |
|  | Lactoc_Q33 (NC_049809) | incomplete | 30 | 28.5 | 35.46 |
|  | Strept_SMP (EF116926.2) | questionable | 90 | 38.8 | 35.12 |
|  | Strept_phiNJ2 (NC_019418) | intact | 150 | 38.9 | 36.52 |
|  | Strept_Str_PAP_1 (NC_28666.1) | intact | 120 | 38.8 | 38.74 |
| Su-19 | Lactoc_bIL311 (NC_002670.1) | incomplete | 50 | 32.9 | 34.91 |
|  | Strept_SMP (EF116926.2) | questionable | 90 | 38.8 | 35.12 |
| Su-20 | Lactoc_Q33 (NC_049809) | incomplete | 30 | 28.5 | 35.46 |
|  | Strept_SMP (EF116926.2) | questionable | 90 | 38.8 | 35.12 |
| Su-21 | Strept_SMP (EF116926.2) | questionable | 90 | 38.8 | 35.12 |
|  | Lactoc_bIL311 (NC_002670.1) | incomplete | 30 | 28.5 | 35.46 |
| Su-22 | Lactoc_Q33 (NC_049809) | incomplete | 50 | 32.9 | 34.91 |
|  | Strept_SMP (EF116926.2) | questionable | 90 | 38.8 | 35.12 |
| Su-23 | Lactoc_lato (NC_004746) | incomplete | 40 | 35.7 | 34.57 |
|  | Strept_7201 (NC_002185) | incomplete | 60 | 43.5 | 34.69 |
|  | Strept_5093 (NC_012753) | intact | 140 | 36.7 | 35.95 |
| Su-24 | Lactoc_Q33 (NC_049809) | incomplete | 50 | 32.9 | 34.91 |
|  | Strept_MM1 (NC_003050.2) | incomplete | 30 | 25.5 | 37.00 |
|  | Strept_SMP (EF116926.2) | questionable | 90 | 38.8 | 35.12 |
| **Reference strains** | | | | | |
| 0140J | Lactoc_bIL310 (NC_002669.1) | incomplete | 20 | 27.1 | 37.61 |
|  | Strept_5093 (NC_012753) | incomplete | 20 | 31.2 | 36.37 |
| EF20 | Lactoc_bIL311 (NC_002670.1) | incomplete | 50 | 40.3 | 36.03 |
